# Impaired Antibody-Independent Immune Response of B Cells in Patients With Acute Dengue Infection

**DOI:** 10.3389/fimmu.2019.02500

**Published:** 2019-10-31

**Authors:** Vinit Upasani, Hoa Thi My Vo, Sivlin Ung, Sothy Heng, Denis Laurent, Rithy Choeung, Veasna Duong, Sopheak Sorn, Sowath Ly, Izabela A. Rodenhuis-Zybert, Philippe Dussart, Tineke Cantaert

**Affiliations:** ^1^Immunology Unit, Institut Pasteur du Cambodge, Institut Pasteur International Network, Phnom Penh, Cambodia; ^2^Department of Medical Microbiology and Infection Prevention, University of Groningen and University Medical Center Groningen, Groningen, Netherlands; ^3^Kantha Bopha Children Hospital, Phnom Penh, Cambodia; ^4^Virology Unit, Institut Pasteur du Cambodge, Institut Pasteur International Network, Phnom Penh, Cambodia; ^5^Epidemiology and Public Health Unit, Institut Pasteur du Cambodge, Institut Pasteur International Network, Phnom Penh, Cambodia

**Keywords:** dengue virus, regulatory B cells (Bregs), immunopathogenesis of dengue, plasmacells, IL-10, B cell subsets

## Abstract

Dengue is a mosquito-borne viral disease caused by dengue virus (DENV). The disease is endemic to more than 100 countries with 390 million dengue infections per year. Humoral immune responses during primary and secondary DENV infections are well-investigated. However, the impact of DENV infection on B cell subsets and their antibody-independent functions are not well-documented. Through this study, we aimed to define the distribution of B cell subsets in the acute phase of DENV infection and characterize the effect of DENV infection on B cell functions such as differentiation into memory and plasma cells and cytokine production. In our cohort of Cambodian children, we observed decreased percentages of CD24^hi^CD38^hi^ B cells and CD27^−^ naïve B cells within the CD19 population and increased percentages of CD27^+^CD38^hi^CD138^+^ plasma cells as early as 4 days post appearance of fever in patients with severe dengue compared to patients with mild disease. Lower percentages of CD19^+^CD24^hi^CD38^hi^ B cells in DENV-infected patients were associated with decreased concentrations of soluble CD40L in patient plasma and decreased platelet counts in these patients. In addition, CD19^+^CD24^hi^CD38^hi^ and CD19^+^CD27^−^ B cells from DENV-infected patients did not produce IL-10 or TNF-α upon stimulation *in vitro*, suggesting their contribution to an altered immune response during DENV infection. In addition, CD19^+^CD27^−^ naïve B cells isolated from dengue patients were refractory to TLR/anti-IgM stimulation *in vitro*, which correlated to the increased expression of inhibitory Fcγ receptors (FcγR) CD32 and LILRB1 on CD19^+^CD27^−^ naïve B cells from DENV-infected patients. Collectively, our results indicate that a defective B cell response in dengue patients may contribute to the pathogenesis of dengue during the early phase of infection.

## Introduction

Dengue is a mosquito-borne viral disease caused by dengue virus (DENV), a positive sense single-stranded RNA virus belonging to the *Flaviviridae* family. The virus is transmitted to humans by mosquitoes of the *Aedes* species, namely, *Aedes aegypti* and *Aedes albopictus* (1). The virus is endemic to more than 100 countries and causes 390 million dengue infections per year, of which one quarter manifests clinical symptoms (2). Clinical presentation of DENV infection can vary from asymptomatic infection with no apparent symptoms or mild dengue fever (DF), which is self-limiting to more severe forms of disease termed dengue hemorrhagic fever (DHF) and dengue shock syndrome (DSS) (3). Around 500,000 people with severe dengue require hospitalization each year with an estimated case fatality rate of 2.5% as reported by the World Health Organization (3). There are four serotypes of dengue virus (DENV1–4) that share 65–80% homogeneity in their genetic sequence and can be distinguished based on serological methods (4). Primary infection with one DENV serotype elicits antibodies with potent protective capacity against homotypic reinfection along with short-lasting cross-protective immunity against other serotypes (1, 2). However, heterologous secondary infections have been shown to be associated with increased severity in patients, resulting in DHF or DSS (5, 6). The exact mechanism of this clinical observation remains to be elucidated. One theory proposed to explain this is termed as antibody-dependent enhancement (ADE) of infection (5, 6). This theory postulates that serotype cross-reactive antibodies can wane over a period of time and upon reaching non-neutralizing concentrations can increase infection by facilitating the FcγR-mediated endocytosis of DENV immune complexes into target cells such as dendritic cells, monocytes, and macrophages (7, 8). Due to ADE and the search for cross-serotype neutralizing antibodies, the humoral immune response to DENV has been a prominent research topic.

Antibodies are produced by terminally differentiated B cells, plasmablasts, and plasma cells. Recent studies have shown that the acute phase of both primary and secondary DENV infections is characterized by a massive increase in the percentages of plasmablasts, especially in patients with severe dengue (9–12). Importantly, however, besides antibody production, B cells have diverse functions and play an important role in antigen presentation (13), inflammation, and production of immunosuppressive cytokines such as IL-10, TGF-β, and IL-35 (14). For example, B cells with regulatory functions, termed Bregs, have important roles in maintenance of tolerance and homeostasis. They have been shown to suppress inflammatory responses in autoimmune disorders (15–17) and viral infections (18–21). Different human B cell subsets have been shown to exhibit regulatory functions such as CD24^hi^CD27^+^ B10 cells (22), CD19^+^CD24^hi^CD27^int^ plasmablasts (23), and CD19^+^CD24^hi^ CD38^hi^ transitional B cells (24) through the production of immunosuppressive cytokines IL-10 and TGF-β. In the context of DENV infection, not much is known about the antibody-independent B cell responses (25, 26).

Hence, we sought to define the distribution of B cell subsets in the early phase of DENV infection and characterize the effect of DENV infection on different B cell functions. We observed increased percentages of developing plasmablasts and plasma cells in dengue-infected patients compared to febrile controls. We detected decreased proportion of CD24^hi^CD38^hi^ transitional B cells/Bregs and CD27^−^ naïve B cells within the CD19+ population during acute DENV infection in patients with severe dengue compared to patients with mild disease, which was associated with decreased CD40L plasma concentrations and decreased platelet counts in these patients. CD19^+^CD24^hi^CD38^hi^ and CD19^+^CD27^−^ naïve B cells from dengue patients did not produce IL-10 cytokine upon stimulation. Moreover, CD19^+^CD27^−^ naïve B cells failed to induce activation markers and antigen presentation molecules upon stimulation, which was paralleled by an increased expression of inhibitory FcγR *ex vivo* in DENV-infected patients. Taken together, our results indicate that a defective B cell response in the early acute phase of infection may contribute to the pathogenesis of DENV infection disease.

## Materials and Methods

### Ethics Statement

Ethical approval for the study was obtained from the National Ethics Committee of Health Research of Cambodia. Written informed consent was obtained from all participants or the guardians of participants under 16 years of age before inclusion in the study.

### Patient Recruitment

Blood samples were obtained from hospitalized children (≥2 years) who presented with dengue-like symptoms at the Kanta Bopha Hospital in Phnom Penh, Cambodia. The time point for collection of blood samples was within 96 h of fever onset at hospital admittance. Patients were classified according to the WHO 1997 criteria upon hospital discharge (3). In total, we recruited 81 dengue-positive patients that were classified for severity (Table 1). Platelet counts were determined by complete blood count at the hospital and available for 59 included children. To detect DENV-specific B cell responses, patients who presented with fever but were negative for DENV were included as controls (*n* = 29) (Table 1). In addition, age- and sex-matched healthy donors were recruited from a cluster-based investigation in Kampong Cham province (*n* = 29) and included for the functional analysis.

**Table 1:** Patient demographics.

	**Dengue patients**			**Febrile controls**	**Healthy donors**
	**Total**	**DF**	**DHF/DSS**		
	**81**	**64**	**17 (9/8)**	**29**	**29**
Age	8.3 ± 3.9	8.3 ± 4.0	8.0 ± 3.5	6.0 ± 4.2	9.2 ± 3.7
M/F ratio	1.1	1.3	0.8	0.9	1.4
Weight (kg)	24.4	25.3	21.1	18.0	27.3
Height (cm)	115.3	114.1	117.4	106.5	123.1
Temperature (°C)	38.0	38.0	37.1	38.4	N/A
Hematocrit (%)	39.3	38.2	42.6	35.1	
Platelets (×10^9^/L)	102.4	120.8	56.9	132.8	
Day of fever, mean (range)	3.5 (2–5)	3.4 (2–5)	4.0 (3–5)	N/A	
DENV1	30	28	2		
DENV2	40	29	11		
DENV3	0	0	0		
DENV4	4	4	0		
NS1+	55	48	7		
PCR+	74	61	11		
Viral load (copies/ml), median (IQR)	17,150 (1,308–438,000)	44,800 (1050–1057000)	7,820 (2,665–51,450)		
Secondary infection	74%	65%	90%		

### Laboratory Diagnosis

Plasma specimens were tested for the presence of DENV using nested qRT-PCR at the Institut Pasteur in Cambodia, the National Reference Center for arboviral diseases in Cambodia (27). NS1 positivity was determined using rapid diagnostic tests (combo test for NS1 and IgM/IgG detection, SD Bioline Dengue Duo kits from Standard Diagnostics, Abbott, Chicago, IL, USA). Anti-DENV IgM was measured with an in-house IgM-capture ELISA (MAC-ELISA), as previously described (28). Samples from patients positive for DENV were further tested with hemagglutination inhibition assay (HIA) to determine primary/secondary DENV infection as per WHO criteria (3).

### B Cell Subset Phenotyping

PBMCs were isolated using Ficoll-Histopaque density gradient centrifugation and were stained *ex vivo* using the following antibodies: CD19 Alexa Fluor 488 (clone HIB19), FcRL4 PE (clone 413D12), CD27 APC/Cy7 (clone O323), CD138 BV421 (clone MI15), IgM PerCp/Cy5.5 (clone MHM-88), CD24 PE/Cy7 (clone ML5), CD38 APC (clone HB7) (all from BioLegend), and IgG BV510 (clone G18-145) (BD Biosciences). Samples were acquired on FACS Canto II (BD Biosciences) and analyzed by FlowJo v10.0 software.

### Culture of B Cells From Healthy Donors and DENV Patients

PBMCs were isolated from healthy donors and dengue patients using Ficoll-Histopaque density gradient centrifugation. All dengue patients used for B cell isolation and further *in vitro* stimulation were classified as DF. Peripheral blood B cells were isolated from PBMCs by positive selection using CD19 MicroBeads (Miltenyi Biotec). B cells were cultured in RPMI 1640 (Gibco) supplemented with 10% FBS (Gibco), penicillin–streptomycin (100 U/ml penicillin, 100 μg/ml streptomycin; Thermo-Fisher), and L-glutamine (2 mM; Invitrogen). To promote cytokine production, B cells were stimulated with CpG (1 μg/ml; Invivogen) and CD40L (0.25 μg/ml; ITS Vietnam) for 48 h. For the last 6 h of incubation, the medium was replaced and cells were incubated with monensin (2 mM; Sigma-Aldrich), PMA (1 mg/ml; Sigma-Aldrich), and ionomycin (1 mg/ml; Sigma-Aldrich) to increase the accumulation of cytokines in the rough endoplasmic reticulum or Golgi complex within the cells and thus improve the sensitivity of intracellular cytokine detection by flow cytometry (24, 29). Cells were harvested after 6 h and surface stained with CD19 BV510 (clone HIB19), CD20 BV421 (clone 2H7), CD27 PerCp/Cy5.5 (clone M-T271), CD24 PE/Cy7 (clone ML5), and CD38 APC (clone HB7) (all from BioLegend) for 30 min. The cells were fixed and permeabilized using TrueNuclear Transcription Buffer Set (BioLegend) as per the manufacturer's protocol and stained intracellularly with antibodies against IL-10 PE and TNF-α APC-Cy7 (all from BioLegend). Supernatants were stored at −80°C for further use. To promote the development of CD138^+^ plasma cells, purified B cells from healthy donor and patient PBMCs were cultured in the presence of CD40L (0.25 μg/ml; ITS Vietnam), IL-2 (1 ng/ml; Peprotech), and IL-21 (50 ng/ml; Peprotech) for 6 days. The cells were harvested and stained with antibodies CD19 PE/Cy7 (clone HIB19), CD20 PerCp/Cy5.5 (clone 2H7), CD27 APC/Cy7 (clone O323), CD138 BV421 (clone MI15), IgM PE (clone MHM-88) (all from BioLegend), and IgG BV510 (clone G18-145) (BD Biosciences). To induce the activation of B cells, B cells isolated from healthy donor and patient PBMCs were cultured in the presence of CpG [1 μg/ml and F(ab')_2_ anti-IgM antibody (4 μg/ml; Jackson ImmunoResearch)] for 2 days. The cells were harvested and stained with HLA-DR PE (clone L243), CD27 PerCp/Cy5.5 (clone M-T271), CD38 APC (clone HB7), TACI PE/Cy7 (clone ML5), CD27 APC/Cy7 (clone O323), CD86 BV421 (clone IT2.2), and CD69 BV510 (clone FN50) (all from BioLegend). The stimulations used for functional assays of B cells are summarized in Supplementary Figure 1. Samples were acquired on FACS Canto II (BD Biosciences) and analyzed by FlowJo v10.0 software.

### Cytometry Bead Assay for Measuring B Cell Cytokines

Concentrations of cytokines IL-6 were quantified in the supernatants from B cells cultured for cytokine production using a LEGENDplex Human B Effector 1/2 Panel immunoassay (BioLegend) as per the manufacturer's instructions. For the detection of cytokines APRIL and CD40L in the plasma of healthy donors and DENV-positive patients, a LEGENDplex Human B cell Activator Panel immunoassay (BioLegend) was used as per the manufacturer's instructions. Samples were acquired using BD FACS Canto II and analyzed using LEGENDplex v7.0 (Vigene Tech, USA) software.

### Statistical Analysis

Statistical analyses were done using GraphPad Prism 7.00 software (GraphPad Software, Inc., La Jolla, CA, USA). Since the data did not pass the criteria for normality using D'Agostino & Pearson normality test, the non-parametric Mann–Whitney *U-*test was used to compare data between two groups or by non-parametric paired Wilcoxon matched pairs signed rank test for paired data. Statistical analysis of data with more than two groups was done using the Kruskal–Wallis test followed by Dunn's post-test for multiple comparisons. For comparing paired samples between three conditions, Friedman's test was used. Correlations were calculated by Spearman analysis. For all analyses, *P* < 0.05 was considered significant.

## Results

### Altered B Cell Subset Distribution in Severe Dengue Infection

In order to understand the B cell responses during early DENV infection and their contribution to disease pathogenesis, we performed detailed phenotyping of B cell subsets in a cohort of pediatric Cambodian patients in the early acute phase of DENV infection. As we aimed to describe the changes in B cell subsets specifically due to dengue infection and not infection in general, we have included a control cohort of children with febrile illness of other origin.

**Figure 1:**
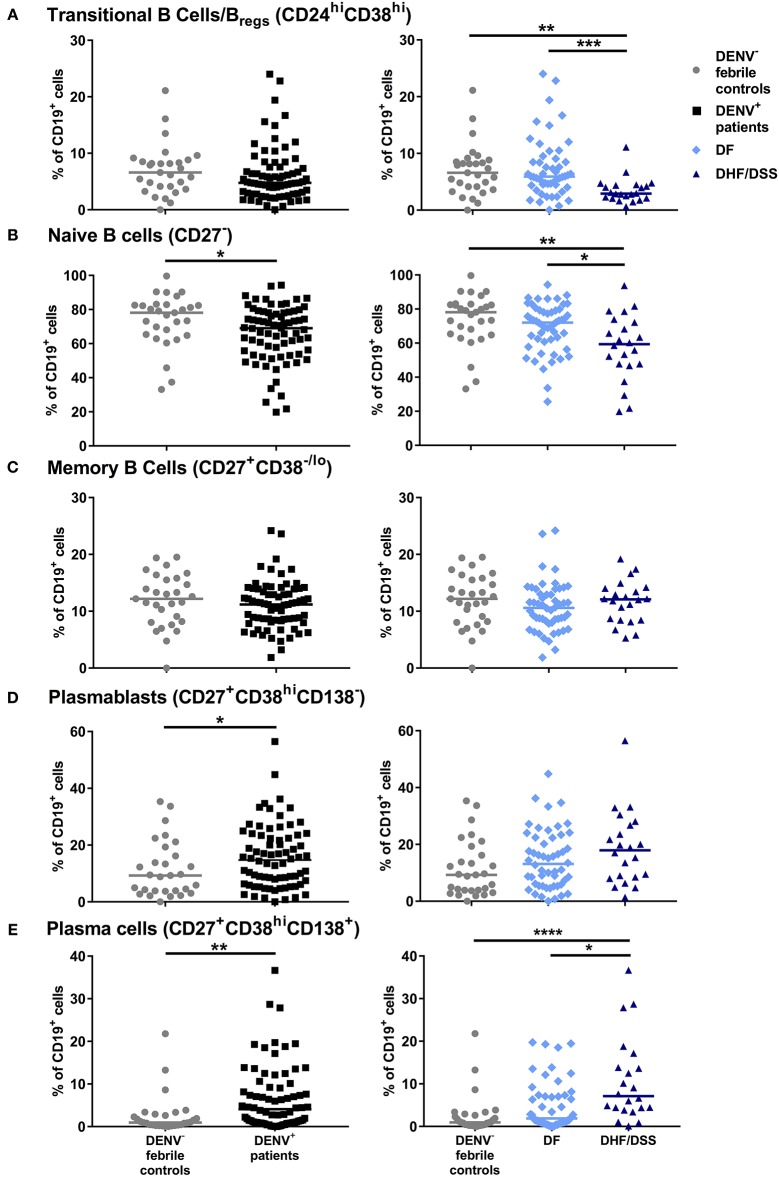
Distribution of B cell subsets during acute DENV infection. PBMCs were stained for B subset-specific markers and gated for each subset. Percentages are reported as percentage of total CD19^+^ cells. Comparison of the percentages of CD24^hi^CD38^hi^ transitional B cells/Bregs **(A)**, CD27^−^ naïve B cells **(B)**, CD27^+^CD38^−/lo^ memory B cells **(C)**, CD27^+^CD38^hi^CD138^−^ plasmablasts **(D)** and CD27^+^CD38^hi^CD138^+^ plasma cells **(E)** in DENV-negative febrile controls (*n* = 29), DENV-positive patients (*n* = 74) (left), and in DF (*n* = 52) and DHF/DSS (*n* = 22) patients (right). Lines indicate median. *P* values were calculated with Mann–Whitney *U*-test for comparing two groups and with Kruskal–Wallis test for comparing more than two groups (*P < 0.05; **P < 0.01, ***P < 0.001; ****P < 0.0001).

The gating strategy for identification of B cell subsets is outlined in Supplementary Figure 2. Frequencies of B cell subsets were compared between patients with confirmed DENV infection (*n* = 74) and those with febrile illness of other origin (*n* = 29) and further within DENV-positive patients between those with dengue fever (DF) (*n* = 52) and severe dengue (DHF/DSS) (*n* = 22) classified according to WHO 1997 criteria (3). The percentages of transitional (CD24^hi^CD38^hi^ cells), which include regulatory, IL-10-producing B cells (Breg), within CD19^+^ B cells, were not different in dengue patients compared to patients with DENV-negative febrile controls. However, percentages of CD24^hi^CD38^hi^ B cells were found to be two-fold lower in patients with DHF/DSS than in patients with DF (median, 2.9 vs 5.9%) (*P* < 0.001) (Figure 1A). The percentage of naïve cells (CD27^−^) was decreased within the CD19^+^ B cell compartment between DENV patients and DENV-negative febrile controls (median, 78.1 vs. 69.0%; *P* < 0.05) and in DHF/DSS patients compared to mild dengue (median, 59.3 vs. 72.0%; *P* < 0.05) (Figure 1B). Whereas, no differences were observed in the memory cells (CD27^+^CD38^−/lo^), the percentages of antibody-secreting plasmablasts (CD27^hi^CD38^hi^CD138^−^) within the CD19^+^ B cell compartment was found to be significantly higher in DENV-positive patients compared to patients with other febrile illness (median, 14.7 vs. 9.3%; *P* < 0.05) (Figures 1C,D), which is surprising given the fact that these children are undergoing acute infections. In parallel, frequencies of CD27^+^CD38^hi^CD138^+^ plasma cells were higher in dengue patients than in febrile controls (median, 4.1 vs. 0.9%; *P* < 0.01) and in DHF/DSS patients compared to DF patients (median, 7.1 vs. 1.9%; *P* < 0.05) (Figure 1E). Given the strong redistribution of the subsets within the CD19 population after infection, we also evaluated the changes in B cell subsets within all total live cells, as we did not have access to the total white blood cell counts for normalization. We observe an increase in naïve, memory B cells and plasma cells within the total live cell compartment in DHF/DSS patients compared to mild dengue patients (Supplementary Figure 3). A decrease of CD19^+^CD24^hi^CD38^hi^ B cells within the total of single cells is not observed; however, this is a small subset and the percentages are very low. Frequencies of circulating immune cell subsets might change day to day during the acute phase of DENV infection. Therefore, we included only patients presenting at the hospital at day 4 of fever for a sub-analysis, which yielded similar differences in B cell subset frequencies as observed in our total cohort (data not shown). As there were more patients in the DHF/DSS group undergoing secondary infection, this could skew our results. Analyzing the different B cell subsets in secondary infected patients only yielded the same results, with significant decreased percentages of CD19^+^CD24^hi^CD38^hi^ B cells (median, 2.9 vs. 5.4%; *P* < 0.01) and naïve B cells (median, 53.9 vs. 68.9%; *P* < 0.05) and significant increased percentages of plasma cells (median, 10.1 vs. 2.9%; *P* < 0.05) in DHF/DSS patients compared to DF patients.

Taken together, the results suggest that the acute phase of DENV infection is characterized by altered B cell subset distribution, which correlates with disease severity.

### Similar Plasma Cell Development in DENV-Infected Individuals and Healthy Individuals

**Figure 2:**
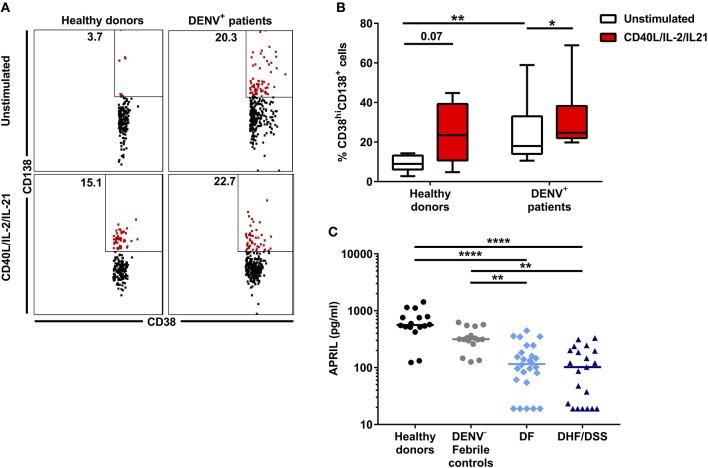
Plasma cell development in DENV-infected patients. B cells isolated from DENV-infected patients (*n* = 6) and healthy donors (*n* = 7) were cultured in the presence of IL-2, IL-21, and CD40L for 6 days. **(A)** Representative dot plots identifying plasma cells based on expression of CD38 and CD138. Similar gating strategy as in Supplementary Figure 2 was used. **(B)** Percentages of CD38^hi^CD138^+^ plasma cells within the CD19^+^CD27^+^ B cell population in healthy donors and DENV patients upon stimulation. Lines and bars indicate median and IQR. *P* values were calculated with Wilcoxon match pairs signed ranked test to compare unstimulated and stimulated conditions and Mann–Whitney *U* test for comparing between healthy donors and DENV^+^ patients. **(C)** Concentrations of APRIL were analyzed in plasma of age-matched healthy donors (*n* = 16), DENV-negative febrile controls (*n* = 16), and patients with acute DENV infection classified according to WHO 1997 guidelines into patients with DF (*n* = 26) and those with DHF/DSS (*n* = 21). Lines indicate median. Kruskal–Wallis test was used to compare multiple groups (*P < 0.05; **P < 0.01; ****P < 0.0001).

As we observed higher concentrations of antibody-secreting cells *in vivo* in dengue-infected patients, we wanted to assess if B cells isolated from dengue patients display an altered response to differentiation signals leading to the development of antibody-secreting cells. Total CD19^+^ cells from dengue patients (*n* = 7) and from age-matched healthy donors (HD, *n* = 8) were isolated by magnetic separation. Due to the low sample volumes available from the pediatric patients, we could not purify different B cell subsets by flow cytometry sorting. Total B cells were cultured *in vitro* with CD40L, IL-2, and IL-21 for 6 days to promote development of plasma cells. Stimulation of total B cells from HD resulted in an increase of the percentage of CD19^+^CD27^+^CD38^hi^CD138^+^ plasma cells compared to unstimulated cells (median, unstimulated: 8.9 vs. stimulated: 23.5%; *P* = 0.07). In parallel, stimulation of B cells from DENV-positive patients also significantly increased the percentage of plasma cells compared to unstimulated cells (median, unstimulated: 18.0 vs. stimulated: 26.5%; *P* < 0.05) (Figures 2A,B). These results suggest that, even though percentages of plasma cells are higher in DENV patients than HD in the unstimulated condition, B cells from both HD and DENV patients have the intrinsic capacity to respond to plasma cell differentiation stimuli. Plasma cells require survival factors such a proliferation inducing ligand (APRIL) for their development and maintenance (30). Therefore, we sought to investigate whether plasma concentrations of APRIL were altered in patients undergoing severe dengue infection. Using a cytometry-based assay, the concentrations of APRIL were determined in patient plasma. The plasma concentrations of APRIL were significantly higher in healthy donors (median: 560.5 pg/ml) and patients with other febrile illness (median: 315.8 pg/ml) compared to DF patients (median: 115.7 pg/ml) or DHF/DSS patients (median: 102.3 pg/ml). Within dengue patients, there was no difference in APRIL plasma concentrations between DF and DHF/DSS (median, DF: 115.7 pg/ml vs. DHF/DSS: 102.3 pg/ml) (Figure 2C). Hence, APRIL cytokine concentrations are lower in patient groups with higher percentages of plasma cells, indicating a possible increased consumption in these patients.

### Decreased Concentrations of CD40L Is Associated With Lower Percentages of CD19^+^*CD*24^hi^CD38^hi^ B Cells in Severe Dengue

**Figure 3:**
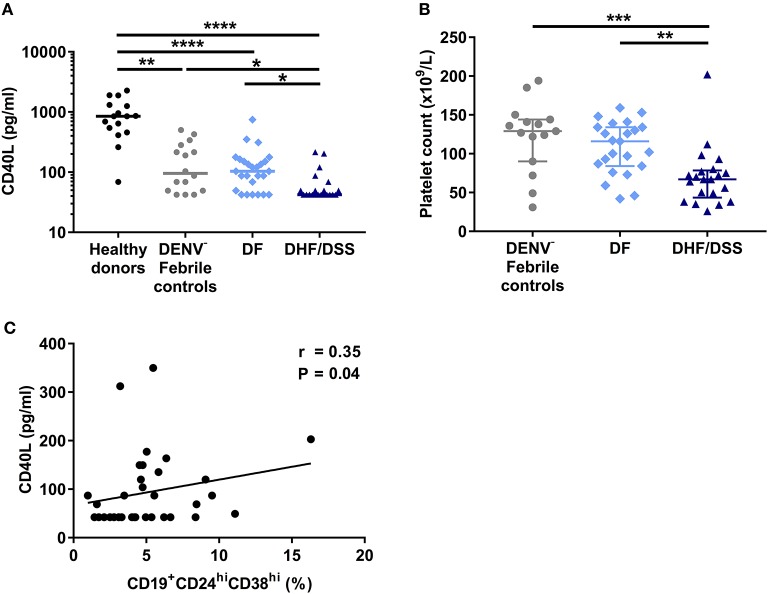
Decrease in sCD40L concentration is associated with increased disease severity and correlates with lower percentages of CD19^+^CD24^hi^CD38^hi^ B cells. **(A)** Concentrations of sCD40L were analyzed in plasma of age-matched healthy donors (*n* = 16), DENV-negative febrile controls (*n* = 16), and patients with acute DENV infection classified according to WHO 1997 guidelines into patients with DF (*n* = 26) and DHF/DSS (*n* = 21). Lines indicate median. *P*-values were calculated with Kruskal–Wallis test for comparing more than two groups. **(B)** Platelet counts in whole blood of DENV-negative febrile controls (*n* = 15), DF patients (*n* = 23), and DHF/DSS patients (*n* = 21) were quantified and shown as platelet count (×10^9^/L). Platelet counts were not available for three DF patients included in this analysis. Lines indicate median. *P*-values were calculated with Kruskal–Wallis test for comparing more than two groups. **(C)** Association between concentrations of CD40L and percentage of CD19^+^CD24^hi^CD38^hi^ B cells in patients with acute DENV infection was calculated using Spearman's correlation (*P < 0.05; **P < 0.01; ***P < 0.001; ****P < 0.0001).

Next, we questioned what factors could contribute to the decreased percentages of CD19^+^CD24^hi^CD38^hi^ B cells observed *ex vivo* in patients with severe dengue compared to mild dengue or patients with other febrile illness. This B cell subset includes regulatory, IL-10-producing B cells (Bregs), which are generated *in vivo* by a combination of type I IFN and CD40L stimulation (31). Therefore, we sought to determine the concentration of soluble CD40L (sCD40L) in HD and patient plasma using a cytometry-based bead assay. HD had significantly higher concentrations of sCD40L (median: 851.3 pg/ml) compared to patients with other febrile illness (median: 95.27 pg/ml, *P* < 0.01) and DF and DHF/DSS patients (*P* < 0.0001). In turn, febrile controls had higher concentrations of sCD40L compared to patients with DHF/DSS (median: 42.06 pg/ml; *P* < 0.05). In addition, the concentration of sCD40L was significantly lower in patients with DHF/DSS with respect to those with DF (median: 103.7 pg/ml; *P* < 0.05) (Figure 3A). sCD40L is mainly produced by activated platelets (32). Indeed, similar to our observations of sCD40L, we observed that patients with other febrile illness had higher platelet counts (median, 127 × 10^9^/L) than patients with DHF/DSS (67 × 10^9^/L; *P* < 0.001) but not patients with DF (116 × 10^9^/L), and we observed decreased platelet counts in patients with DHF/DSS compared to those with DF (*P* < 0.01) (Figure 3B). Moreover, in dengue patients, lower concentrations of sCD40L correlated with lower percentages of CD19^+^CD24^hi^CD38^hi^ B cells (*R* = 0.35; *P* < 0.05) (Figure 3C). Hence, lower concentrations of sCD40L may contribute to the decreased percentages of CD19^+^CD24^hi^CD38^hi^ B cells observed in patients with severe dengue.

### B Cells From Dengue Patients Produce Less Cytokines Upon *in vitro* Stimulation

**Figure 4:**
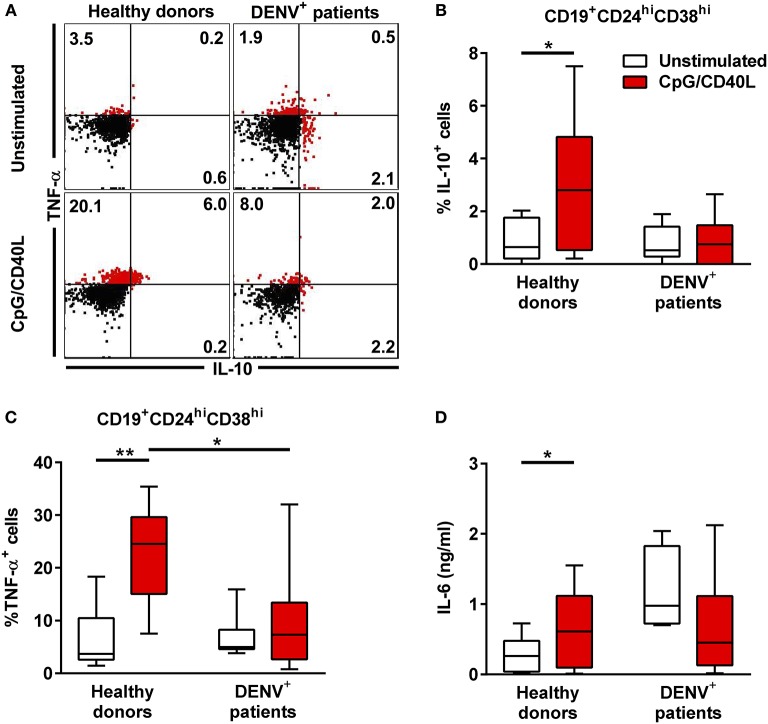
Cytokine response in B cells during acute DENV infection. Total CD19^+^ B cells isolated from DENV-infected patients (*n* = 7) and healthy donors (*n* = 8) were stimulated with CD40L and CpG for 48 h. **(A)** Representative dot plot of HD and DENV^+^ B cells that were gated in CD19^+^CD24^hi^CD38^hi^ B cells and analyzed for IL-10 and TNF-α staining. **(B,C)** Summary of the data showing % of IL10 and TNF-α positive cells within the CD19^+^CD24^hi^CD38^hi^ gate. **(D)** IL-6 in B cell supernatants were quantified using a cytometry-based bead assay. Concentrations are shown in ng/ml. Bars and lines represent median and IQR. *P*-values were calculated with Wilcoxon match pairs signed ranked test to compare unstimulated and stimulated conditions and by Mann–Whitney *U*-test for comparing between healthy donors and DENV^+^ patients (*P < 0.05; **P < 0.01).

To understand in greater detail the antibody-independent functions of B during DENV infection, we investigated the cytokine production of total CD19^+^ B cells isolated from DENV-positive patients (*n* = 8) and age-matched healthy donors (*n* = 7) after *in vitro* stimulation with CD40L and CpG for 48 h, which is known to induce cytokine secretion in B cells. During the last 6 h of incubation, PMA and ionomycin were added to the cells along with protein transport inhibitors, and the cells were stained intracellularly for IL-10 and TNF-α (Figure 4A). Gating on CD19^+^CD24^hi^CD38^hi^ B cells, which include Breg cells, we observed that these cells produced both IL-10 and TNF-α upon stimulation in healthy donors (median, % IL-10^+^: 2.80%; % TNF-α: 24.55%). In contrast, CD19^+^CD24^hi^CD38^hi^ from DENV-infected patients did not upregulate the production of both TNF-α and IL-10 (median, % IL-10^+^: 0.75%; % TNF-α^+^: 7.33%) (Figures 4B,C). The same significant differences were observed gating on CD19^+^CD27^−^ naïve cells (Supplementary Figures 4A,C), with similar trends for CD19^+^CD27^+^ memory cells, although the variability was higher in this subset (Supplementary Figures 4B,D). Within the same line, healthy donor CD19^+^ B cells secreted IL-6 in the cell culture supernatant upon stimulation, whereas B cells isolated from DENV patients did not produce IL-6 upon stimulation (Figure 4D). Hence, reduced B-cell-derived cytokine production during the acute phase of DENV infection might contribute to disease development.

### B Cells From Dengue Patients Are Refractory to Toll-like Receptor (TLR) Stimulation

**Figure 5:**
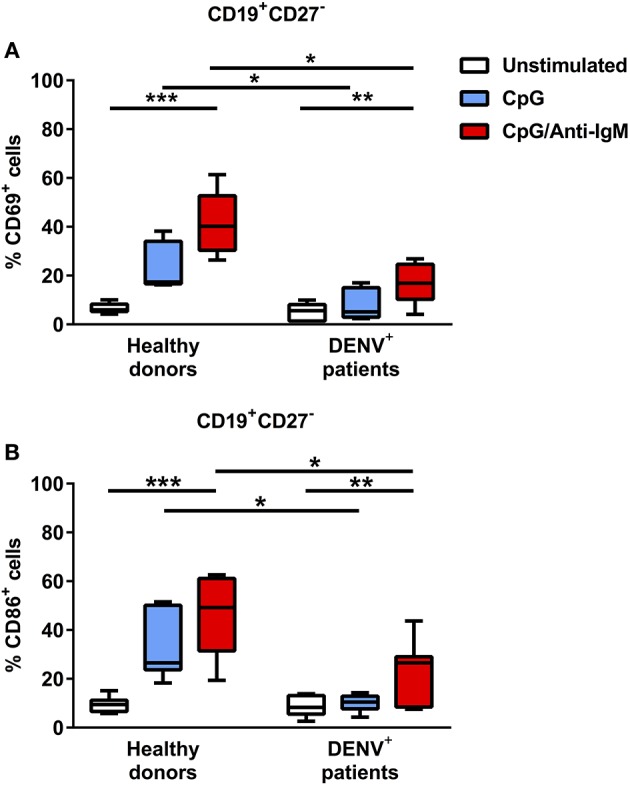
B cells from DENV-infected patients are refractory to TLR stimulation but can be activated upon co-stimulation with BCR and TLR agonists. B cells isolated from DENV-infected patients (*n* = 7) and healthy donors (*n* = 7) were stimulated with CpG alone or with anti-IgM antibody for 48 h. Percentages of CD69^+^ **(A)** and CD86^+^ **(B)** cells were evaluated in the CD19^+^CD27^−^ naïve B cell population. Lines represent median and IQR. *P*-values were calculated with Friedman's test for comparing paired samples between three groups and Mann–Whitney *U* test for comparing between healthy donors and DENV^+^ patients (*P < 0.05; **P < 0.01; ***P < 0.001).

Since we observed decreased cytokine responses in B cells of dengue patients compared to healthy donors after TLR agonist/CD40L stimulation, we aimed to investigate if B cells from dengue patients can be sufficiently activated by TLR stimulation in general. Gating on CD19^+^CD27^−^ naïve B cells, we observed that activation after TLR stimulation is decreased in dengue patients compared to healthy donors as measured by the lower percentage of early activated, CD69^+^ naïve B cells or naïve B cells expressing CD86, a co-stimulatory protein for T cell activation during antigen presentation (median CD69^+^, 5.1% vs. 17.4%; *P* < 0.05) (median CD86^+^, 8.3 vs. 26.5%; *P* < 0.05) (Figures 5A,B). In addition, strong stimulation via B-cell receptor (BCR) and TLR only partially restored the activation of the B cells derived from dengue patients. Here, the percentage of CD69^+^ and CD86^+^ naïve B cells remained significantly lower in B cells from dengue patients with respect to healthy donors even upon stimulation via BCR and TLR (median CD69^+^, 16.9 vs 40.2%; *P* < 0.05) (median CD86^+^, 26.5 vs 49.2%; *P* < 0.05) (Figure 5). Overall, the results suggest that B cells isolated from dengue patients during the acute phase of infection are functionally impaired.

### B Cells From Dengue Patients Express Higher Percentage of Inhibitory Receptors

**Figure 6:**
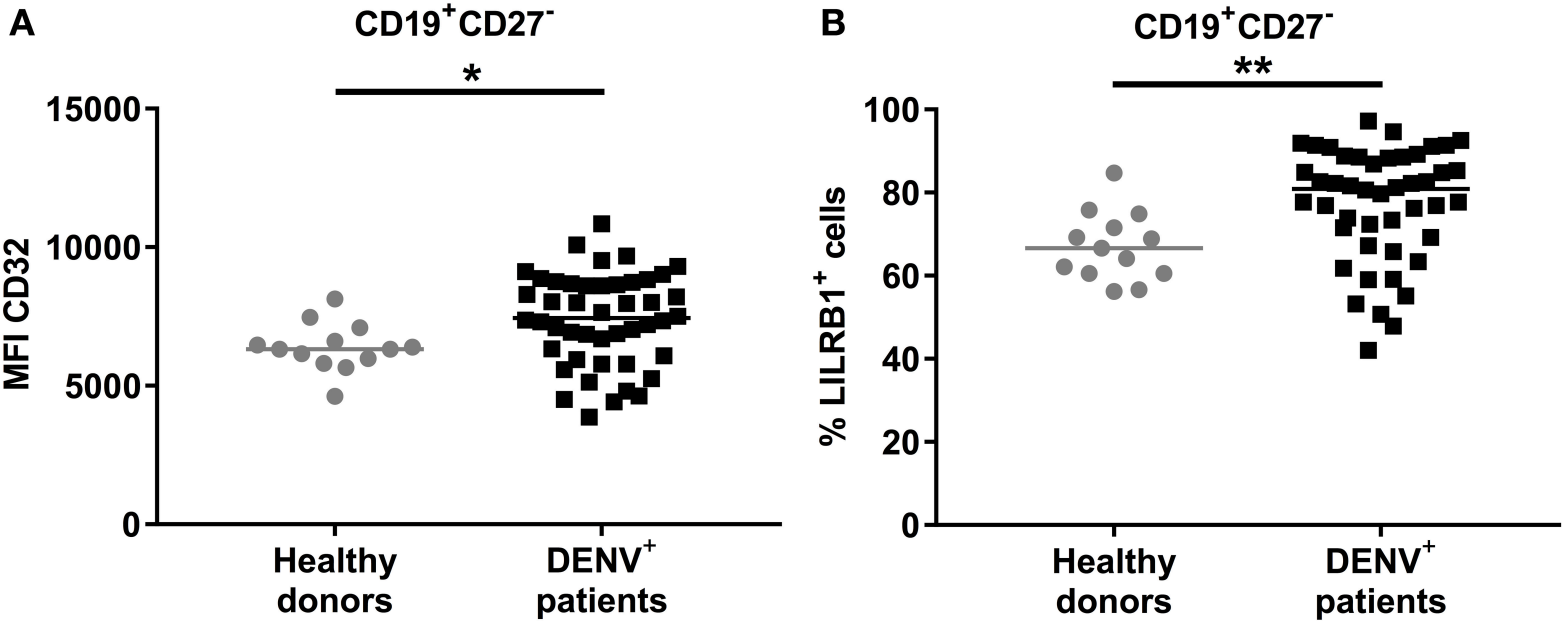
Expression of inhibitory Fc receptors on B cells. CD19^+^ B cells isolated from PBMCs of healthy donors (*n* = 13) and DENV-infected patients (*n* = 46) were stained direct *ex vivo* for CD19, CD27, CD32, and LILRB1. Cells were gated on CD19^+^CD27^−^ naïve B cells. **(A)** MFI of CD32 expression and **(B)** percentage of cells expressing LILRB1 was determined in naïve B cells. Lines indicate median. *P*-values were calculated with Mann–Whitney *U*-test (*P < 0.05; **P < 0.01).

We aimed to understand why B cells derived from dengue patients are impaired in their activation and cytokine production. B cell activation is regulated by both activating and inhibitory receptors such as CD32B (FcγRIIB) and LILRB1 (33, 34). Therefore, expression of these two receptors was investigated directly *ex vivo* in CD19^+^CD27^−^ naïve B cells from DENV-positive patients. The expression of CD32B was found to be significantly upregulated on naïve B cells from DENV-positive patients compared to those from healthy donors (median, 7439 vs. 6321; *P* < 0.05) (Figure 6A). Similarly, the percentage of LILRB1-expressing cells was increased in naïve B cells from DENV-positive patients compared to healthy donors (80.6 vs. 66.6%; *P* < 0.01) (Figure 6B). Atypical B cells, such as B cells expressing FcRL4, are included within the CD27^−^ B cell population and express high percentages of inhibitory receptors (35, 36). However, percentages of CD19^+^CD27^−^FcRL4^+^ cells were low in dengue-infected patients (median: 0.9 %, interquartile range: 0.2–1.7%, Supplementary Figure 5). Thus, these results suggest that during the acute phase of DENV infection, naïve B cells upregulate the expression of inhibitory Fc receptors, and this may contribute to the reduced B cell activation and cytokine production.

## Discussion

In this study, we characterized the B cell subsets and the antibody-independent functions of B cells in a cohort of Cambodian pediatric patients in the early phase of DENV infection. We observed that the distribution of B cell subsets was significantly altered in DENV-positive patients compared to children undergoing febrile illness, which correlated with disease severity within the dengue-infected patients. This is demonstrated by an increase in the percentages of plasma cells and decrease in Bregs/transitional and naïve B cells in DHF/DSS patients. In addition, we observed a functionally impaired B cell response in dengue patients defined by the absence of cytokine production upon stimulation. Naïve B cells of DENV-infected patients showed a lower activation status and upregulation of inhibitory receptors on the cell surface.

Previous studies have demonstrated that DENV infection is characterized by a marked increase in the percentages of plasmablasts and plasma cells in patients at days 4–7 post appearance of symptoms (9, 11, 37). Here, we report a significantly higher percentage of plasmablasts and plasma cells in dengue patients compared to patients with other febrile illness, even earlier during the course of disease, within 96 h of onset of fever. These data suggest that DENV induces an increase in plasma cells independent of the normal response to infection. We also observed that the percentages of plasma cells were significantly higher in patients with DHF/DSS, even during acute infection and irrespective of immune status, both within the CD19^+^ population and within the total population of live cells. Unfortunately, we were unable to analyze the absolute cell counts, which would have been informative given the redistribution of most B cell subsets after infection. In addition, we were unable to analyze multiple time points during the acute phase of infection in order to follow the evolution of the B cell frequencies. We previously reported an increased expression of genes related to plasma cell development in hospitalized dengue patients compared to acute DENV-infected, asymptomatic patients (38). Altogether, these observations further support the involvement of plasmablasts/plasma cells in the immunopathogenesis of the disease. However, it is still unknown what drives this steep increase in plasmablast/plasma cell development in dengue infection. Here, we demonstrate that B cells from DENV-infected individuals respond the same to *in vitro* T-cell independent differentiation conditions to plasma cells as healthy donor B cells. In addition, there are no differences in APRIL plasma concentrations, a cytokine important for plasma cell development and survival, between mild and severe disease, even though concentrations are lower compared to DENV-febrile controls and healthy donors, suggesting consumption of the cytokine by the developing plasma cells. Further studies are needed to define the mechanisms underlying the steep and early increase of plasma cells in severe dengue cases.

We observed here a two-fold decrease in CD24^hi^CD38^hi^ cells within the CD19^+^ B cell population in patients undergoing severe dengue disease (DHF or DSS). This B cell subset includes a population of Bregs due to their ability to produce substantial amounts of IL-10, an immunoregulatory cytokine (39, 40). This subset of B cells is often reduced in numbers in patients with autoimmune disorders (15–17, 41) or upon infections (18–21) but has not been studied previously in the context of DENV infection. CD24^hi^CD38^hi^ Bregs are generated by a combination of type I IFN and CD40L stimulation (31). Type I IFN plays an important role in DENV infection. However, type I IFN circulates at only trace levels in the serum during acute infection and is difficult to reliably determine its concentrations (32, 42, 43). We show here decreased plasma concentrations of sCD40L in patients with severe dengue. These data are in line with previous findings, where decreased serum concentrations of sCD40L were observed in adult DENV-infected patients with plasma leakage (44). sCD40L is primarily produced by platelets and DENV-2 infection can induce the production of sCD40L by platelets (45, 46). Indeed, we observed platelet counts to be significantly lower in patients with severe dengue. Hence, the decrease in sCD40L in severe disease is probably due to a lowering in platelets, which might result in the decrease of CD19^+^CD24^hi^CD38^hi^ cells we observe in severe disease. Indeed, studies have shown that patients with immune thrombocytopenia tend to have lower percentages of CD24^hi^CD38^hi^ Bregs (47, 48).

Besides decreased percentages of CD24^hi^CD38^hi^ transitional B cells/Bregs and CD27^−^ naïve B cells in severe dengue infection, we also demonstrate here the inability of B cells isolated from dengue patients to produce IL-10 and TNF-α upon TLR/CD40 stimulation. The absence of IL-10 production may lead to a defective feedback mechanism and reduced suppression of the inflammatory response and thus might contribute to the excessive immune activation and cytokine storm observed in these patients (49).

B cells isolated from dengue patients were refractory to TLR9 stimulation, as we did not observe an upregulation of activation markers or co-stimulatory markers required for antigen presentation in naïve B cells after *in vitro* stimulation. These data are in parallel to what has been observed in myeloid and plasmacytoid dendritic cells in Colombian individuals (50). Whether this might be due to a lower expression profile of TLR9 in B cells, and/or interference of DENV with the TLR9 pathway remains to be investigated (50, 51). Strong B-cell stimulation through both the TLR and BCR pathway induces B-cell activation in B cells from DENV-infected individuals, albeit to a lower extent than in healthy donors. Of note, the B cells of DENV-infected patients are not refractory to CD40 and cytokine stimulation, as demonstrated by the similar increase in plasma cells observed in B cells from DENV-infected patients and healthy donors. A limitation of the study design of all the *in vitro* functional assays is that these have been performed with total CD19^+^ purified cells, rather than with pure B cell subpopulations as we did not obtain sufficient number of cells for flow cytometry cell sorting. Our studied cohort is composed of pediatric patients; hence, only small sample volumes were available for analysis.

The threshold of B cell activation is regulated not only by BCR and/or TLR but also by inhibitory receptors such as CD32B (FcγRIIB) and LILRB1 (33, 34). We observed increased expression of CD32B and LILRB1 on CD19^+^CD27^−^ B cells directly *ex vivo* during the early phase of dengue infection. CD32B is a key negative regulator of BCR signaling. Upon co-ligation with the immune complex along with the B cell receptor (BCR), CD32B inhibits the immune response by blocking the BCR signaling pathway (52, 53). Another Fc-like receptor with inhibitory function, LILRB1, is also expressed on B cells. It binds to MHC I on antigen-presenting cells and transduces a negative signal that inhibits the immune response (33). Interestingly, both receptors are implicated in the mechanism of ADE during DENV infection and are known to be exploited by DENV to regulate anti-viral responses (54–57). Alternatively, atypical B cells expressing high percentages of inhibitory receptors might also be increased within CD27^−^ B cells. Even though percentages of FcRL4^+^ B cells were low in naïve B cells of dengue positive patients, we did not evaluate other subsets of atypical B cells such as FcRL5^+^, CD11c^+^T-bet^+^, T-bet^hi^CD85j^hi^, or CD21^−/lo^CD27^−^ B cells, which might affect our results (35, 36, 58, 59).

In conclusion, we observed decreased percentages of CD24^hi^CD38^hi^ B cells and CD27^−^ naïve B cells within the CD19 population and increased percentages of CD27^+^CD38^hi^CD138^+^ plasma cells correlating with disease severity within 4 days post appearance of fever symptoms. CD19^+^CD24^hi^CD38^hi^ and CD19^+^CD27^−^ B cells from dengue patients were refractory to TLR stimulation *in vitro*, resulting in decreased B-cell-specific IL-10 and TNF-α cytokine production, which correlates to an increased expression of inhibitory FcγR *in vivo* in naïve B cells from DENV-infected patients. Taken together, our results indicate a defective B cell response in dengue patients that may contribute to the pathogenesis of DENV during the acute phase of infection. These data emphasize the importance of antibody-independent B cell functions in the early phase of disease development after dengue infection.

## Data Availability Statement

The raw data supporting the conclusions of this manuscript will be made available by the authors, without undue reservation, to any qualified researcher.

## Ethics Statement

The studies involving human participants were reviewed and approved by National Ethics Committee for Health Research, Cambodia. Written informed consent to participate in this study was provided by the participants' legal guardian/next of kin.

## Author Contributions

VU, HV, SU, and RC performed the experiments and interpreted the data. SH, DL, SS, and SL included patients and coordinated clinical data management. VD and PD, and TC coordinated experiments and interpreted data. IR-Z, PD, and TC designed the study. VU, HV, PD, IR-Z, and TC wrote the manuscript.

### Conflict of Interest

The authors declare that the research was conducted in the absence of any commercial or financial relationships that could be construed as a potential conflict of interest.

## Supplementary Material

Supplementary Figure 1 *In vitro* cell culture conditions used for functional studies on B cells from healthy donors and DENV-infected patients.

Supplementary Figure 2 Representative gating strategy used to define B cell subsets. Total B cells (A; CD19^+^) were further gated for Bregs/transitional B cells (B1; CD19^+^CD24^high^CD38^high^). Based on expression of CD19 and CD27, total B cells were further gated as naïve B cells (B2; CD19^+^CD27^−^). CD27^+^ B cells were gated as memory B cells (B3; CD19^+^CD27^+^CD38^−/low^) and antibody secreting cells (CD19^+^CD27^+^CD38^hi^) based on CD38 expression. Antibody secreting cells were classified as plasmablasts (B4; CD19^+^CD27^+^CD38^high^CD138^−^), and plasma cells (B5; CD19^+^CD27^+^CD38^high^CD138^+^) based on CD138 expression.

Supplementary Figure 3 PBMCs were stained for B subset-specific markers and gated for each subset. Data are reported as percentage of total live cells. Comparison of the percentages of CD24^hi^CD38^hi^ transitional B cells/Bregs **(A)**, CD27^−^ naïve B cells **(B)**, CD27^+^CD38^−/lo^ memory B cells **(C)**, CD27^+^CD38^hi^CD138^−^ plasmablasts **(D)** and CD27^+^CD38^hi^CD138^+^ plasma cells **(E)** in DENV-negative febrile controls (*n* = 29), DENV-positive patients (*n* = 74) (left) and in DF (*n* = 52) and DHF/DSS (*n* = 22) patients (right). Lines indicate median. *P*-values were calculated with Mann–Whitney *U-*test for comparing two groups and with Kruskal–Wallis test for comparing more than two groups (**P* < 0.05; ***P* < 0.01, ****P* < 0.001).

Supplementary Figure 4 Total CD19^+^ B cells isolated from DENV-infected patients (*n* = 7) and healthy donors (*n* = 8) were stimulated with CD40L and CpG for 48 h. **(A,C)** Summary of the data showing % of IL10 and TNF-α positive cells within the CD19^+^CD27^−^ gate. **(B,D)** Summary of the data showing % of IL10 and TNF-α positive cells within the CD19^+^CD27^+^ gate. Bars and lines represent median and IQR. *P*-values were calculated with Wilcoxon match pairs signed ranked test to compare unstimulated and stimulated conditions and by Mann–Whitney *U*-test for comparing between healthy donors and DENV^+^ patients (**P* < 0.05; ***P* < 0.01).

Supplementary Figure 5 PBMCs were stained for B subset-specific markers and gated to determine the expression of FcRL4. **(A)** CD19^+^ B cells were gated based on the expression of CD27 and FcRL4 to determine the percentage of CD19^+^CD27^−^FcRL4^+^ B cells. **(B)** Comparison of the percentages of FcRL4^+^ cells within the CD19^+^CD27^−^ naïve B cell population in DENV-negative febrile controls (*n* = 20) and DENV-positive patients (*n* = 44). Lines indicate median. Mann–Whitney *U*-test was used for comparing the two groups.

## References

[1] Guzman MG Kouri G. Dengue and dengue hemorrhagic fever in the Americas: lessons and challenges. J Clin Virol. (2003) 27:1–13. 10.1016/S1386-6532(03)00010-6 12727523

[2] Bhatt S Gething PW Brady OJ Messina JP Farlow AW Moyes CL. The global distribution and burden of dengue. Nature. (2010) 496:504–7. 10.1038/nature12060 23563266 PMC3651993

[3] World Health Organization Dengue Guidelines for Diagnosis, Treatment, Prevention and Control. Geneva: World Health Organization Press (1997).

[4] Simmons CP Farrar JJ Vinh Chau N Wills B. Dengue. N Engl J Med. (2012) 366:1423–32. 10.1056/NEJMra1110265 22494122

[5] Katzelnick LC Montoya M Gresh L Balmaseda A Harris E. Antibody-dependent enhancement of severe dengue disease in humans. Science. (2017) 358:929–32. 10.1126/science.aan6836 29097492 PMC5858873

[6] Salje H Cummings DAT Rodriguez-Barraquer I Katzelnick LC Lessler J Klungthong C. Reconstruction of antibody dynamics and infection histories to evaluate dengue risk. Nature. (2018) 557:719–23. 10.1038/s41586-018-0157-4 29795354 PMC6064976

[7] Ng JKW Zhang SL Tan HC Yan B Maria Martinez Gomez J Tan WY. First experimental *in vivo* model of enhanced dengue disease severity through maternally acquired heterotypic dengue antibodies. PLoS Pathog. (2014) 10:e1004031. 10.1371/journal.ppat.1004031 24699622 PMC3974839

[8] Halstead SB O'Rourke EJ. Dengue viruses and mononuclear phagocytes. I. Infection enhancement by non-neutralizing antibody. J Exp Med. (1977) 146:201–17. 10.1084/jem.146.1.201 406347 PMC2180729

[9] Wrammert J Onlamoon N Wittawatmongkol O Wilson PC Pattanapanyasat K Angkasekwinai N. Rapid and massive virus-specific plasmablast responses during acute dengue virus infection in humans. J Virol. (2012) 86:2911–8. 10.1128/JVI.06075-11 22238318 PMC3302324

[10] Priyamvada L Cho A Onlamoon N Zheng NY Huang M Kovalenkov Y. B cell responses during secondary dengue virus infection are. J Virol. (2016) 90:5574–85. 10.1128/JVI.03203-15 27030262 PMC4886779

[11] Garcia-Bates TM Cordeiro MT Nascimento EJ Smith AP Soares de Melo KM McBurney SP. Association between magnitude of the virus-specific plasmablast response and disease severity in dengue patients. J Immunol. (2012) 190:80–7. 10.4049/jimmunol.1103350 23203929 PMC3529775

[12] Zompi S Montoya M Pohl MO Balmaseda A Harris E. Dominant cross-reactive B cell response during secondary acute dengue virus infection in humans. PLoS Negl Trop Dis. (2012) 6:e1568. 10.1371/journal.pntd.0001568 22448292 PMC3308930

[13] Rodríguez-Pinto D. B cells as antigen presenting cells. Cell Immunol. (2005) 238:67–75. 10.1016/j.cellimm.2006.02.005 16574086

[14] Fillatreau S Sweenie CH McGeachy MJ Gray D Anderton SM. B cells regulate autoimmunity by provision of IL-10. Nat Immunol. (2002) 3:944–50. 10.1038/ni833 12244307

[15] Hilgenberg E Shen P Dang VD Ries S Sakwa I Fillatreau S. Interleukin-10 producing B cells and the regulation of immunity. Curr Top Microbiol Immunol. (2014) 380:69–92. 10.1007/978-3-662-43492-5_4 25004814

[16] Mauri C Gray D Mushtaq N Londei M. Prevention of arthritis by interleukin 10–producing B cells. J Exp Med. (2003) 197:489–501. 10.1084/jem.20021293 12591906 PMC2193864

[17] Silverman GJ Srikrishnan R Germar K Goodyear CS Andrews KA Ginzler EM. Genetic imprinting of autoantibody repertoires in systemic lupus erythematosus patients. Clin Exp Immunol. (2008) 153:102–16. 10.1111/j.1365-2249.2008.03680.x 18510544 PMC2432104

[18] Das A Ellis G Pallant C Lopes AR Khanna P Europe PMC Funders Group IL-10 producing regulatory B cells in the pathogenesis of chronic HBV infection. J Immunol. (2013) 189:3925–35. 10.4049/jimmunol.1103139 PMC3480715 22972930

[19] Gong Y Zhao C Zhao P Wang M Zhou G Han F. Role of IL-10-producing regulatory B cells in chronic hepatitis B virus infection. Dig Dis Sci. (2015) 60:1308–14. 10.1007/s10620-014-3358-1 25260658

[20] Siewe B Keshavarzian A Martinson J Kazmi N French AL Landay A. Regulatory B cell frequency correlates with markers of HIV disease progression and attenuates anti-HIV CD8 + T cell function *in vitro*. J Leukoc Biol. (2013) 93:811–8. 10.1189/jlb.0912436 23434518 PMC3629440

[21] Liu J Huibner S Gregor A Zhao H Cao JC Yue FY. IL-10-producing B cells are induced early in HIV-1 infection and suppress HIV-1-specific T cell responses. PLoS ONE. (2014) 9:e89236. 10.1371/journal.pone.0089236 24586620 PMC3931714

[22] Iwata Y Matsushita T Horikawa M DiLillo DJ Yanaba K Venturi GM. Characterization of a rare IL-10-competent B-cell subset in humans that parallels mouse regulatory B10 cells. Blood. (2011) 117:530–41. 10.1182/blood-2010-07-294249 20962324 PMC3031478

[23] Matsumoto M Baba A Yokota T Nishikawa H Ohkawa Y Kayama H. Interleukin-10-producing plasmablasts exert regulatory function in autoimmune inflammation. Immunity. (2014) 41:1040–51. 10.1016/j.immuni.2014.10.016 25484301

[24] Blair PA Flores-Borja F Noreña LY Isenberg DA Rawlings DJ Mauri C CD19+CD24hiCD38hi B cells exhibit regulatory capacity in healthy individuals but are functionally impaired in systemic lupus erythematosus patients. Immunity. (2010) 32:129–40. 10.1016/j.immuni.2009.11.009 20079667

[25] Castañeda DM Salgado DM Narváez CF. B cells naturally induced during dengue virus infection release soluble CD27, the plasma level of which is associated with severe forms of pediatric dengue. Virology. (2016) 497:136–45. 10.1016/j.virol.2016.07.014 27467579

[26] Perdomo-celis F Romero F Salgado DM Vega R. Identification and characterization at the single-cell level of cytokine-producing cells in children with dengue. J Infect Dis. (2018) 217:1472–80. 10.1093/infdis/jiy053 29390091

[27] Hue KD Tuan TV Thi HT Bich CT Anh HH Wills BA. Validation of an internally controlled one-step real-time multiplex RT-PCR assay for the detection and quantitation of dengue virus RNA in plasma. J Virol Methods. (2011) 177:168–73. 10.1016/j.jviromet.2011.08.002 21843553 PMC4347661

[28] Duong V Deubel V Lorn Try P Vong S Ly S Chroeung N. Clinical and virological factors influencing the performance of a NS1 antigen-capture assay and potential use as a marker of dengue disease severity. PLoS Negl Trop Dis. (2011) 5:e1244. 10.1371/journal.pntd.0001244 21811645 PMC3139664

[29] Bankó Z Pozsgay J Szili D Tóth M Gáti T Nagy G. Induction and differentiation of IL-10–producing regulatory B cells from healthy blood donors and rheumatoid arthritis patients. J Immunol. (2017) 198:1512–20. 10.4049/jimmunol.1600218 28087671

[30] Cassese G Arce S Hauser AE Lehnert K Moewes B Mostarac M. Plasma cell survival is mediated by synergistic effects of cytokines and adhesion-dependent signals. J Immunol. (2003) 171:1684–90. 10.4049/jimmunol.171.4.1684 12902466

[31] Menon M Blair PA Isenberg DA Mauri C. A regulatory feedback between plasmacytoid dendritic cells and regulatory B cells is aberrant in systemic lupus erythematosus. Immunity. (2016) 44:683–97. 10.1016/j.immuni.2016.02.012 26968426 PMC4803914

[32] Seo YJ Kim GH Kwak HJ Nam JS Lee HJ Suh SK. Validation of a HeLa Mx2/Luc reporter cell line for the quantification of human type I interferons. Pharmacology. (2009) 84:135–44. 10.1159/000235158 19684437

[33] Karnell JL Dimasi N Karnell FG Fleming R Kuta E Wilson M. CD19 and CD32b differentially regulate human B cell responsiveness. J Immunol. (2014) 192:1480–90. 10.4049/jimmunol.1301361 24442430 PMC3918864

[34] Merlo A Saverino D Tenca C Battini L Ciccone E Fais F. Inhibitory receptors CD85j, LAIR-1, and CD152 down-regulate immunoglobulin and cytokine production by human B lymphocytes. Clin Vaccine Immunol. (2005) 12:705–12. 10.1128/CDLI.12.6.705-712.2005 15939744 PMC1151979

[35] Ehrhardt GRA Davis RS Hsu JT Leu C Ehrhardt A Cooper MD The inhibitory potential of FcRL4 on memory B cells. Proc Natl Acad Sci USA. (2003) 100:13489–94. 10.1073/pnas.1935944100 14597715 PMC263841

[36] Jourdan M Robert N Cren M Thibaut C Duperray C Kassambara A. Characterization of human FcRL4-positive B cells. PLoS ONE. (2017) 12:e0179793. 10.1371/journal.pone.0179793 28636654 PMC5479562

[37] Balakrishnan T Leo YS Devi S Ooi EE Hibberd ML Flamand M. Dengue virus activates polyreactive, natural IgG B cells after primary and secondary infection. PLoS ONE. (2011) 6:e29430. 10.1371/journal.pone.0029430 22216280 PMC3245273

[38] Simon-Loriere E Duong V Twafik A Ung S Ly S Casademont I. Increased adaptive immune responses and proper feedback regulation protect against clinical dengue. Sci Transl Med. (2019) 9:eaal5088. 10.1126/scitranslmed.aal5088 28855396

[39] Palanichamy A Barnard J Zheng B Owen T Quach T Looney RJ. novel human transitional B cell populations revealed by B cell depletion therapy. J Immunol. (2009) 182:5982–93. 10.4049/jimmunol.0801859 19414749 PMC2746373

[40] Sims GP Ettinger R Shirota Y Yarboro CH Illei GG Lipsky PE. Identification and characterization of circulating human transitional B cells. Blood. (2005) 105:4390–8. 10.1182/blood-2004-11-4284 15701725 PMC1895038

[41] Mavropoulos A Simopoulou T Varna A Liaskos C Katsiari CG Bogdanos DP. Breg cells are numerically decreased and functionally impaired in patients with systemic sclerosis. Arthrit Rheumatol. (2016) 68:494–504. 10.1002/art.39437 26414243

[42] Hua J Kirou K Lee C Crow MK. Functional assay of type I interferon in systemic lupus erythematosus plasma and association with anti-RNA binding protein autoantibodies. Arthritis Rheum. (2006) 54:1906–16. 10.1002/art.21890 16736505

[43] Rentsch MB Zimmer G A vesicular stomatitis virus replicon-based bioassay for the rapid and sensitive determination of multi-species type I interferon. PLoS ONE. (2011) 6::e25858 10.1371/journal.pone.0025858 21998709 PMC3187809

[44] Romano CM Heitman J Keating SM Moraes Ferreira F Cerdeira Sabino E Lanteri MC. Serum from dengue virus-infected patients with and without plasma leakage differentially affects endothelial cells barrier function *in vitro*. PLoS ONE. (2017) 12:e0178820. 10.1371/journal.pone.0178820 28586397 PMC5460851

[45] Jin Y Nonoyama S Morio T Imai K Ochs HD Mizutani S. Characterization of soluble CD40 ligand released from activated Platelets. J Med Dent Sci. (2001) 48:23–7. 10.11480/jmds.480104 12160239

[46] Mosso-Pani M Sánchez-Torres L Salazar M Corona-de la Peña N Castro-Mussot M Núñez-Avellaneda D. Dengue virus induces the release of sCD40L and changes in levels of membranal CD42b and CD40L molecules in human platelets. Viruses. (2018) 10:357. 10.3390/v10070357 29976871 PMC6071282

[47] Li X Haider MA Evangelista J Yazdanbakhsh K Zhong H Boulad N. Defective regulatory B-cell compartment in patients with immune thrombocytopenia. Blood. (2012) 120:3318–25. 10.1182/blood-2012-05-432575 22859611 PMC3476542

[48] Yazdanbakhsh K Zhong H Bao W. Immune dysregulation in immune thrombocytopenia. Semin Hematol. (2013) 50 (Suppl. 1):S63–7. 10.1053/j.seminhematol.2013.03.011 23664520 PMC3658172

[49] Rothman AL. Immunity to dengue virus: a tale of original antigenic sin and tropical cytokine storms. Nat Rev Immunol. (2011) 11:532–43. 10.1038/nri3014 21760609

[50] Torres S Hernández JC Giraldo D Arboleda M Rojas M Smit JM. Differential expression of toll-like receptors in dendritic cells of patients with dengue during early and late acute phases of the disease. PLoS Negl Trop Dis. (2013) 7:e2060. 10.1371/journal.pntd.0002060 23469297 PMC3585035

[51] Lai J Wu C Ke P Huang C Liu S Luo S. Infection with the dengue RNA virus activates TLR9 signaling in human dendritic cells. EMBO Rep. (2018) 19:e46182. 10.15252/embr.201846182 29880709 PMC6073071

[52] Nimmerjahn F Ravetch JV. Fcγ receptors as regulators of immune responses. Nat Rev Immunol. (2008) 8:34–47. 10.1038/nri2206 18064051

[53] Muta T Kurosaki T Misulovin Z Sanchez M Nussenzweig MC Ravetch JV. A 13-amino-acid motif in the cytoplasmic domain of Fc gamma RIIB modulates B-cell receptor signalling. Nature. (1994) 368:70–3. 10.1038/368070a0 8107887

[54] Ong EZ Chan KR Ooi EE. Viral manipulation of host inhibitory receptor signaling for immune evasion. PLOS Pathogens. (2016) 12:e1005776. 10.1371/journal.ppat.1005776 27584579 PMC5008827

[55] Boonnak K Slike BM Donofrio GC Marovich MA Human Fc RII cytoplasmic domains differentially influence antibody-mediated dengue virus infection. J Immunol. (2013) 190:5659–65. 10.4049/jimmunol.1203052 23616574 PMC3659957

[56] Chan KR Zhang SL Tan HC Chan YK Chow A Lim AP. Ligation of Fc gamma receptor IIB inhibits antibody-dependent enhancement of dengue virus infection. Proc Natl Acad Sci USA. (2011) 108:12479–84. 10.1073/pnas.1106568108 21746897 PMC3145677

[57] Chan KR Ong EZ Tan HC Zhang SL Zhang Q Tang KF. Leukocyte immunoglobulin-like receptor B1 is critical for antibody-dependent dengue. Proc Natl Acad Sci USA. (2014) 111:2722–7. 10.1073/pnas.1317454111 24550301 PMC3932915

[58] Li H Borrego F Nagata S Tolnay M. Fc Receptor-like 5 expression distinguishes two distinct subsets of human circulating tissue-likememory B cells. J Immunol. (2016) 196:4064–74. 10.4049/jimmunol.1501027 27076679

[59] Knox JJ Buggert M Kardava L Seaton KE Eller MA Canaday DH. T-bet+ B cells are induced by human viral infections and dominate the HIV gp140 response. JCI Insight. (2017) 2:92943. 10.1172/jci.insight.92943 28422752 PMC5396521

