# Supplementary figures and images for: Impaired Antibody-Independent Immune Response of B Cells in Patients With Acute Dengue Infection
Source: Front Immunol. 2019 Oct 31;10:2500. doi: 10.3389/fimmu.2019.02500 (PMC6834554; doi:10.3389/fimmu.2019.02500)

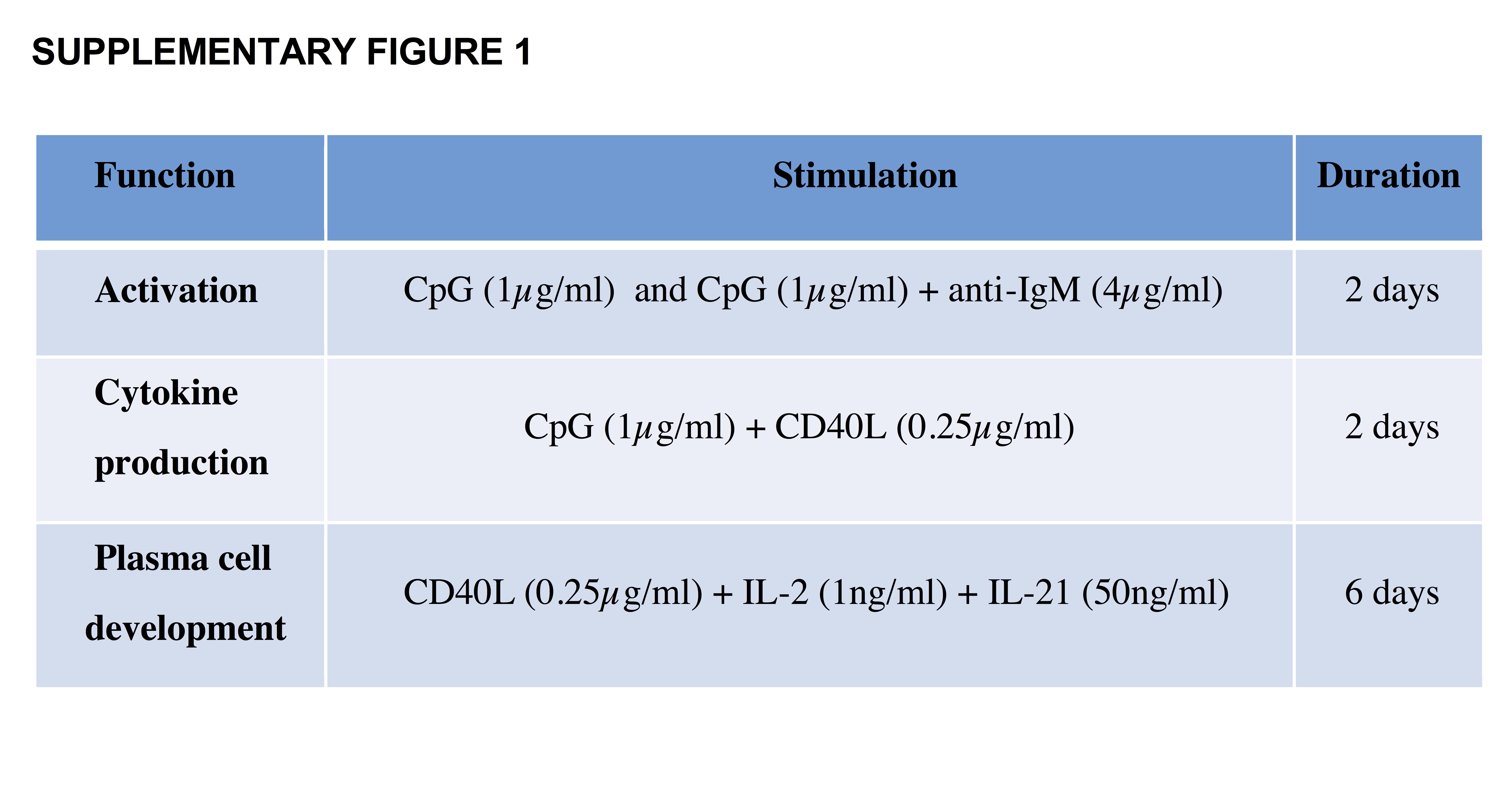

Supplement: Supplementary Figure 1 — In vitro cell culture conditions used for functional studies on B cells from healthy donors and DENV-infected patients. [file Image_1.JPEG]

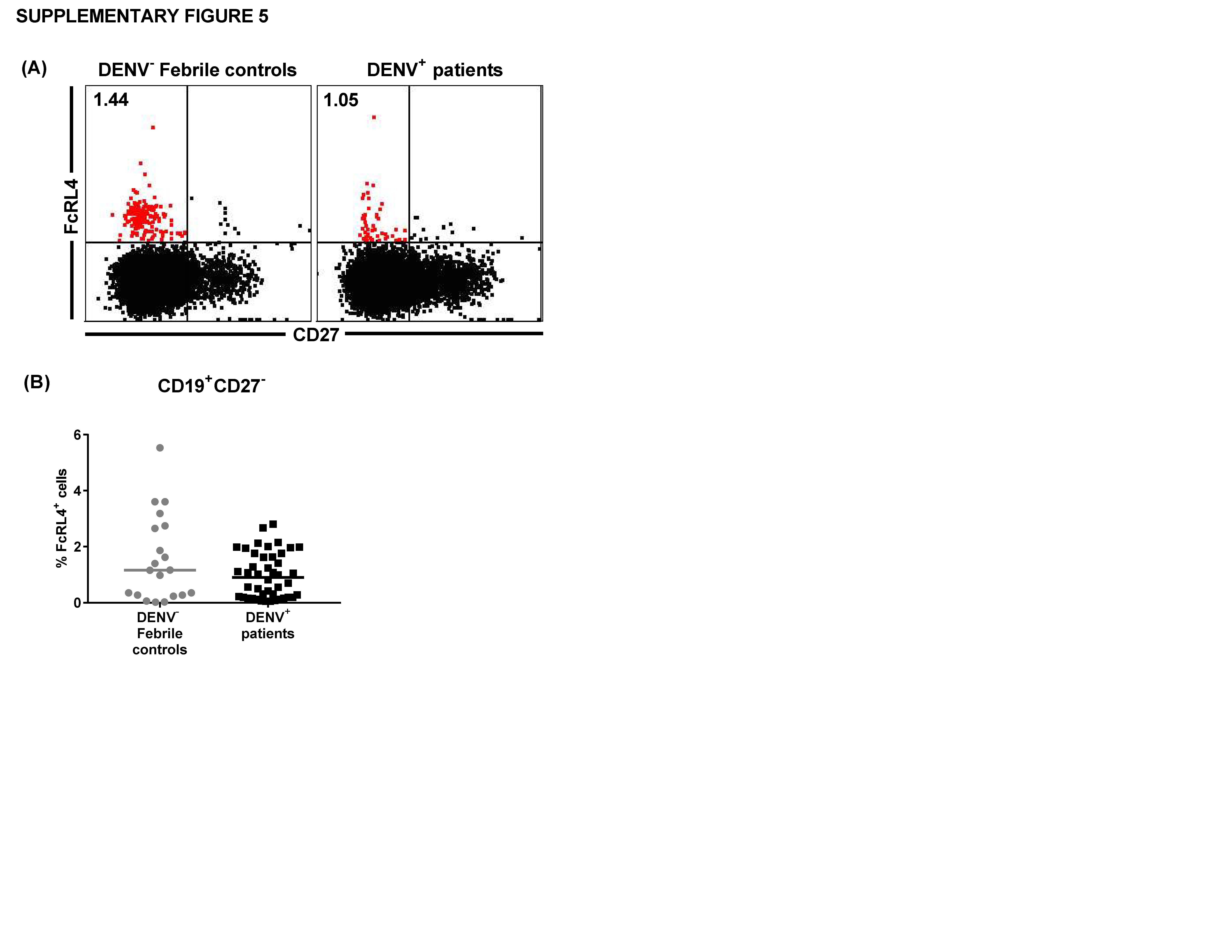

Supplement: Supplementary Figure 5 — PBMCs were stained for B subset-specific markers and gated to determine the expression of FcRL4. (A) CD19+ B cells were gated based on the expression of CD27 and FcRL4 to determine the percentage of CD19+CD27−FcRL4+ B cells. (B) Comparison of the percentages of FcRL4+ cells within the CD19+CD27− naïve B cell population in DENV-negative febrile controls (n = 20) and DENV-positive patients (n = 44). Lines indicate median. Mann–Whitney U-test was used for comparing the two groups. [file Image_5.jpeg]
